# New dimensions of PD-1/PD-L1 inhibitor combination therapy in cancer treatment: current advances and future perspectives

**DOI:** 10.3389/fimmu.2025.1616872

**Published:** 2025-08-27

**Authors:** Cheng Tong, Yue Wu, Renzhao Wu

**Affiliations:** ^1^ First People’s Hospital of Linping District, Hangzhou, China; ^2^ The First Affiliated Hospital of Zhejiang Chinese Medical University (Zhejiang Provincial Hospital of Chinese Medicine), Hangzhou, Zhejiang, China; ^3^ The Key Laboratory of Research and Development of Chinese Medicine of Zhejiang Province, Zhejiang Academy of Traditional Chinese Medicine, Tongde Hospital of Zhejiang Province, Hangzhou, Zhejiang, China

**Keywords:** immune checkpoint, combination therapy, PD-1/PD-L1, traditional Chinese medicine (TCM), immune-related adverse events (IRAE)

## Abstract

The treatment of tumors remains one of the most challenging issues in modern medicine. For a long time, surgical intervention and radiochemotherapy were the primary methods employed to combat tumors; however, the therapeutic outcomes often fell short of expectations. Immunotherapy offers a promising alternative by enhancing the patient’s immune system’s ability to recognize and eliminate tumor cells while minimizing damage to normal cells and tissues. This advancement has brought new hope for cancer patients. In recent years, significant progress has been made in tumor immunotherapy with immune checkpoint inhibitors (ICIs), particularly Programmed Cell Death Protein-1 (PD-1)/Programmed Cell Death-Ligand 1 (PD-L1) inhibitors. An increasing number of researchers have discovered that combination therapy involving PD-1/PD-L1 inhibitors alongside chemotherapeutic agents or other types of ICIs yields more pronounced effects compared to monotherapy. Nevertheless, there remains a considerable risk of developing resistance and experiencing various adverse events. Traditional Chinese medicine (TCM) is frequently utilized as an adjunctive treatment for tumors due to its potential to enhance overall immune function in patients. Recent studies indicate that combining TCM with PD-1/PD-L1 inhibitors can significantly improve median survival times for patients, undoubtedly providing new directions for cancer treatment, however, there remains a lack of sufficient large-sample prospective controlled studies to provide evidence supporting the combined treatment of traditional Chinese medicine and immune checkpoint inhibitors for tumors. This study summarizes recent research on the combined use of PD-1/PD-L1 inhibitors with chemotherapeutic drugs, other ICIs, or TCM in cancer therapy. The aim is to explore their synergistic mechanisms and clinical application value while drawing greater attention from scholars to the significant value of traditional Chinese medicine in the combined treatment strategies for tumors. Additionally, this research provides insights into future prospects for anti-tumor research within TCM.

## Introduction

Currently, cancer remains one of the most challenging diseases to cure. The treatment of cancer has undergone a lengthy evolution; prior to the 21st century, surgical resection was the predominant method employed for cancer treatment. However, since most cancers are already advanced or have metastasized to adjacent tissues and organs by the time they are diagnosed, surgery often cannot achieve complete eradication. Consequently, targeted therapeutic approaches such as radiotherapy and chemotherapy have been developed (1). Chemotherapy agents can directly inhibit the DNA synthesis of tumor cells, thereby interfering with their growth and differentiation. Radiotherapy employs high doses of high-energy radiation to damage the DNA of tumor cells, achieving a similar effect. It is worth noting that for an extended period, even up to the present day, radiochemotherapy has significantly enhanced the survival rates of certain cancer patients due to its pronounced antitumor effects, establishing it as one of the first-line treatment modalities for cancer. However, the cytotoxic effects associated with radiochemotherapy also impact normal cells, often disrupting the homeostasis of the patient’s immune system and leading to a series of severe complications. Consequently, further exploration into safe and effective therapeutic approaches has become a crucial direction in anticancer research.

Research indicates that during tumor development, T cells undergo exhaustion. The T cells that should be activated exhibit a high expression of certain surface molecules that inhibit their activation. These include Programmed Cell Death Protein-1 (PD-1), Cytotoxic T-Lymphocyte Antigen 4 (CTLA-4), and Lymphocyte Activation Gene-3 (LAG-3). Collectively, these molecules are referred to as immune checkpoints (2). Research has shown that immune checkpoints play a crucial role in the regulation of immune homeostasis and are highly expressed on the surfaces of tumor cells as well as various immune cell types (3). The binding of immune checkpoints to their ligands can inhibit certain signaling pathways, serving the purpose of maintaining immune tolerance. However, tumor cells often exploit these mechanisms to evade immune surveillance, a phenomenon commonly referred to as “immune evasion” (4). In this research context, the investigators discovered that immune checkpoint inhibitors (ICIs) can prevent the binding of immune checkpoints to their ligands or interfere with their functional activities post-binding, thereby achieving anti-tumor effects. This led to the introduction of the concept of immunotherapy. Immunotherapy enhances the body’s ability to recognize tumor cells and utilize the immune system to eliminate them. It has garnered widespread attention and is gradually becoming a primary approach in anti-tumor treatment (5) (6). The PD-1/PD-L1 inhibitors, as a class of ICIs, play a significant role in restoring T cell-mediated anti-tumor immune responses. They have demonstrated remarkable efficacy in the treatment of various cancer types, including melanoma, lung cancer, and gastrointestinal tumors (7, 8). However, clinical evidence indicates that the use of PD-1/PD-L1 inhibitors as monotherapy for tumor treatment is associated with notable limitations. These include a low objective response rate, an increased likelihood of secondary resistance development, and potential risks of adverse toxic effects (9–11). This review systematically examines the critical clinical challenges inherent in PD-1/PD-L1 inhibitor monotherapy and highlights recent advances in combination therapies involving chemotherapy regimens, other ICIs, and active components of traditional Chinese medicine (TCM). The aim is to clarify the synergistic mechanisms underlying enhanced efficacy and reduced toxicity across different combination strategies, as well as their translational significance in clinical practice.

This review conducted a systematic search in PubMed, China National Knowledge Infrastructure (CNKI), and Wanfang Database. The search strategies employed included: “(PD-1/PD-L1 inhibitors) AND (combination)”, “(Bevacizumab) AND (combination)”, “(Nivolumab) AND (combination)”, “(Atezolizumab) AND (combination)”, “(PD-1/PD-L1 inhibitors) AND (traditional Chinese medicine)”, “(PD-1/PD-L1 inhibitors) AND (decoction)”, “(PD-1/PD-L1 inhibitors) AND (injection solution)”, “(PD-1/PD-L1 inhibitors) AND (compound preparations)”, “(ICIs) AND (chemotherapy)” etc. The keywords used included: tumor, cancer, combination therapy, traditional Chinese medicine, ICIs, among others. Studies published from 2015 to 2025 that met the criteria were retrieved.

## Interactions between PD-1 and PD-L1, and their inhibitors

PD-1 is an immune checkpoint receptor that is expressed on the surface of various immune cells, including macrophages, dendritic cells, B lymphocytes, and tumor-specific activated T cells (12, 13). PD-L1 is one of the natural receptors for PD-1, primarily expressed on antigen-presenting cells and tumor cells (14). When researchers explore the occurrence and development of tumors, it is essential to address the concept of “T cell exhaustion.” Under sustained antigenic stimulation, activated specific CD8+ T cells gradually lose their potent effector functions. This process leads to the expression of various inhibitory receptors, such as PD-1, resulting in the inability of the immune system to eliminate antigens effectively (15).

Recent studies have demonstrated that the primary mechanism of anti-tumor activity associated with PD-1/PD-L1 inhibitors involves PD-1^+^ TCF-1^+^ stem-like CD8^+^ T cells, specifically the exhausted CD8^+^ T cells (T_pex_). During tumor progression, blocking PD-1 pathway leads to providing proliferative burst of (transitory) effector-like CD8 T cells from stem-like CD8 T cells, which subsequently differentiate into effector-like CD8^+^ T cells. These effector-like cells engage in the tumor immune microenvironment, exhibiting characteristics typical of effectors and migrating to key sites of tumor development. Ultimately, thereby exerting control over tumor progression (15–17).

Furthermore, research has revealed that the activation of PD-L1 leads to the phosphorylation of PD-1 by protein tyrosine kinases. Subsequently, Src homology region 2 domain-containing phosphatase (SHP-2) is recruited, resulting in the dephosphorylation of T cell receptors (TCR). This process significantly inhibits TCR signaling pathways (18). The PD-1 receptor can transmit inhibitory signals through the immunoreceptor tyrosine-based inhibition motif and the immunoreceptor tyrosine-based activation motif, thereby inhibiting the RAS/MEK/ERK and PI3K/AKT signaling pathways. This results in a reduction of T cell proliferation and cytokine secretion (19).

**Figure 1:**
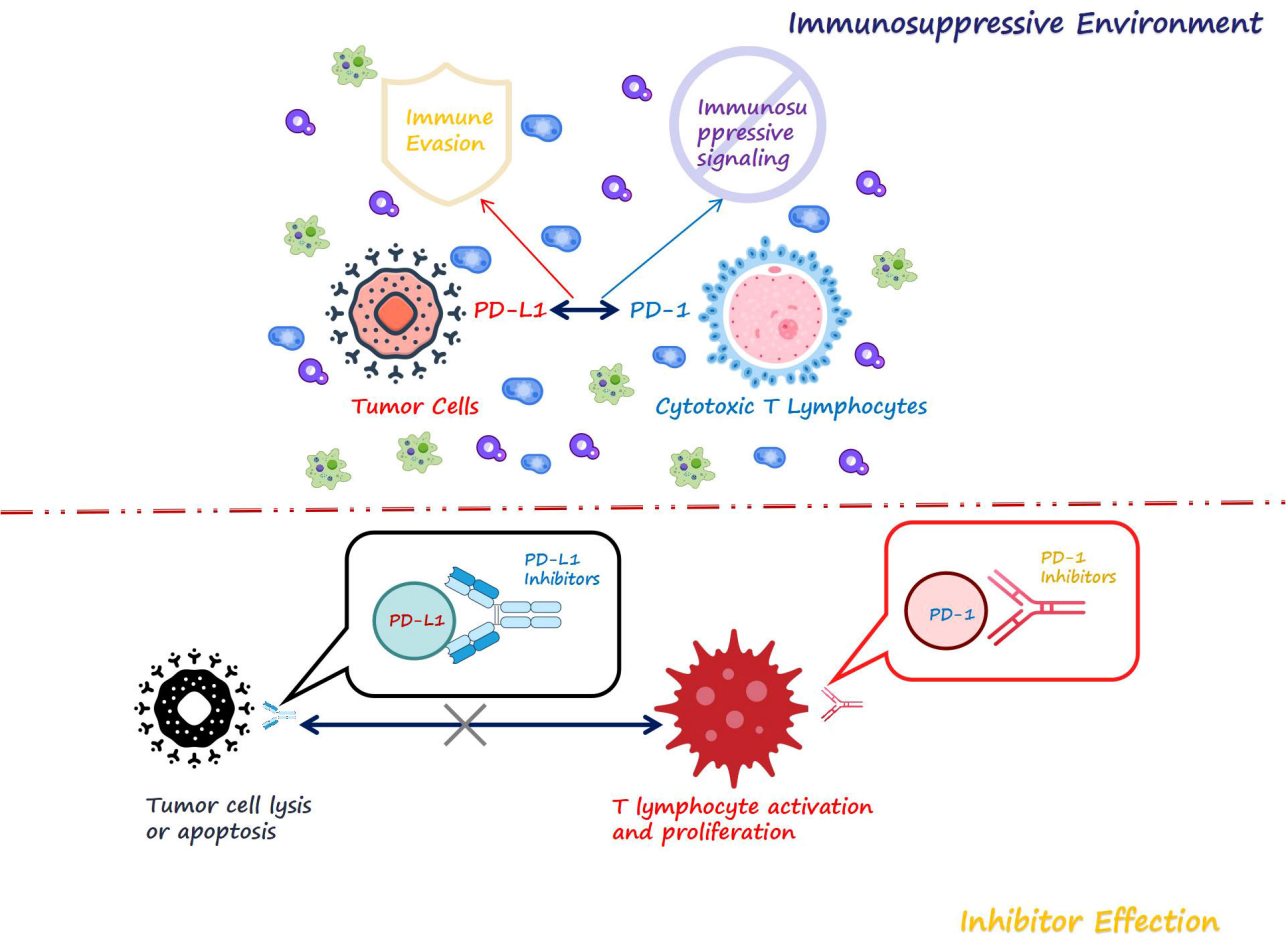
The immune microenvironment and the mechanism of ICIs.

The PD-1 and PD-L1 inhibitors can bind to PD-1 and PD-L1, respectively, thereby inhibiting their interaction (**Figure 1**). When PD-1/PD-L1 inhibitors exert their effects, they lead to a failure in the phosphorylation of PD-1 while suppressing the dephosphorylation of TCR. As a result, activation signals generated by antigen-presenting cells can be effectively transmitted downstream through TCR pathways. This stimulation promotes T cell proliferation and differentiation, thereby enhancing the immune system’s surveillance capabilities and facilitating the destruction of tumor cells (20).

## Challenges of monotherapy with PD-1/PD-L1 inhibitors

Despite the fact that currently approved PD-1/PD-L1 inhibitors have benefited some patients and significantly improved survival rates, 50%-60% of patients still exhibit low responsiveness or no response at all (21). Furthermore, numerous studies have reported on the resistance to PD-1/PD-L1 inhibitors as well as immune-related adverse events (irAEs) (**Table 1**). In immunotherapy, many patients, despite meeting the treatment criteria, exhibit an inherent lack of responsiveness to the drugs due to their tumor cells’ primary resistance. For instance, a deficiency in immunogenicity is one of the potential reasons for the development of resistance during monotherapy (22). A study on metastatic urothelial carcinoma has demonstrated that transforming growth factor β (TGF-β) can inhibit T lymphocytes, particularly CD8+ T lymphocytes, thereby affecting the antitumor efficacy of PD-L1 inhibitors (23). Additionally, research has indicated that the intensity of IFNγ signaling within tumor cells is positively correlated with their responsiveness to PD-1 inhibitors. Different phenotypes of tumor cells exhibit varying sensitivities to PD-1 inhibitors, which may be related to the strength of IFNγ signaling. In other words, low levels of IFNγ signaling could be one of the contributing factors leading to primary resistance (24). Tumor cells can secrete certain inhibitors, such as VEGF or IL-10, which lead to a reduction in the number of normal mature dendritic cells. As a result, effector T cells are not effectively activated during the antigen presentation process (25). Some patients initially exhibit favorable therapeutic responses; however, over the course of treatment, they gradually develop tolerance to the medication, resulting in tumor progression and acquired resistance. The mechanism of acquired drug resistance is highly complex and may involve multiple factors, including the tumor immune microenvironment (TIME), genetic mutations, and alterations in the gut microbiota. Tumor cells, together with various immune cells such as T cells, regulatory T cells (Tregs), myeloid-derived suppressor cells (MDSCs), and stromal cells, collaboratively establish an immunosuppressive TIME, which protects tumor cells from immune-mediated clearance (26, 27). Proteomics and RNA analysis have revealed that collagen in tumors exerts its effects through the LAIR1 receptor, leading to the exhaustion and functional impairment of CD8+ T cells. This phenomenon results in patient resistance to PD-1 inhibitors. However, reducing the deposition of collagen in tumor cells can restore this resistance. Consequently, collagen and LAIR1 are considered potential biomarkers for predicting the efficacy of PD-1 inhibitors (28). In non-small cell lung cancer (NSCLC), the Stk11/Lkb1 gene is subject to deletions and mutations, which contribute to the resistance of tumor patients to PD-1/PD-L1 inhibitors (29). The resistance to PD-1/PD-L1 inhibitors can also be attributed to abnormalities in the composition of gut microbiota. Research has shown that patients who are resistant to PD-1/PD-L1 inhibitors often experience alterations in their gut microbiome structure due to antibiotic use, which disrupts the homeostasis of gut flora. In animal experiments, fecal transplants from mice sensitive to PD-1/PD-L1 inhibitors into the intestines of resistant mice resulted in a significant restoration of sensitivity to these inhibitors. Metagenomic analysis indicated that this phenomenon is associated with the relative abundance of Akkermansia muciniphila (30).

**Table 1:** The ICI monotherapy dilemma.

Events	Mechanisms	References
Low responsiveness	Epigenetic defects and Signaling defects	(21)
Primary resistance	Deficiency in immunogenicity	(22)
	Low levels of IFNγ signaling	(24)
	Inhibitors secreted by tumor cells	(25)
Secondary resistance	Tumor immune microenvironment	(28)
	Genetic mutations	(29)
	Alterations in the gut microbiota	(30)
irAEs	Uncontrolled inflammation	(41, 42)
	Shared antigens	(43)

Long-term use of ICIs continuously stimulates the immune system, which may lead to the occurrence of irAEs (31, 32). Numerous studies have demonstrated that the use of PD-1/PD-L1 inhibitors alone in the treatment of various types of cancer patients is associated with a significantly elevated risk of irAEs, with this incidence typically reaching 20% to 30% (33–35), including risks associated with drug toxicity-related mortality and inflammatory responses in target organs (36, 37). For patients who have previously experienced irAEs, the risk of developing irAEs again upon re-treatment with PD-1/PD-L1 inhibitors rises to 34.2% (38). This risk is also associated with factors such as the type of medication used and the specific tumor type involved (39, 40). The uncontrolled inflammation and shared antigens may be the primary causes of irAEs. Researchers have observed that following the administration of PD-1/PD-L1 inhibitors, there is a significant infiltration of T cells into both tumor tissues and even normal tissues. This phenomenon can lead to the release of numerous inflammatory factors, resulting in adverse reactions (41). Patients with melanoma often experience gastrointestinal adverse reactions such as diarrhea or colitis following the use of PD-1 inhibitors. Notably, serum levels of IL-10 and IL-17 in these patients are frequently significantly elevated, with serum IL-10 also serving as a potential predictor for melanoma recurrence (42). In patients with NSCLC, nine antigens identical to those found in human skin tissue have been identified on the surface of tumor cells. This suggests that while patients undergo monotherapy immunotherapy, normal skin tissue may also be affected (43).

It is evident that, despite the groundbreaking advancements achieved with PD-1/PD-L1 inhibitors in tumor immunotherapy, monotherapy still presents certain limitations. To enhance efficacy and overcome resistance, exploring combination therapy strategies has become an inevitable trend.

## Therapeutic strategies based on the combination of PD-1/PD-L1 inhibitors and chemotherapeutic agents

Although PD-L1 inhibitors may demonstrate significant efficacy in patients with advanced NSCLC who exhibit high levels of PD-L1 expression (44), they can also accelerate tumor progression in a subset of these patients (45). Chemotherapy agents can delay the growth of tumor cells by stimulating immune responses, inhibiting DNA replication, and interfering with cellular metabolism and the cell cycle (46, 47). Therefore, chemotherapy remains a high-priority treatment option for most cancers. In recent years, clinical trials have been conducted to investigate the efficacy of combining PD-1/PD-L1 inhibitors with chemotherapy drugs, aiming to determine whether this combination therapy can achieve improved therapeutic outcomes. In experiments involving lung cancer gene mice treated with low-dose Apatinib in combination with PD-L1 inhibitors, it was observed that the concurrent administration of low-dose Apatinib and anti-PD-L1 significantly delayed tumor growth. The researchers confirmed that low-dose Apatinib enhances the infiltration of CD8+ T cells while reducing the recruitment of tumor-associated macrophages within the tumor microenvironment, thereby optimizing its conditions (48). Triple-negative breast cancer is characterized by high heterogeneity, aggressive behavior, and a lack of specific therapeutic targets, which often results in limited efficacy of chemotherapy. This has posed a significant challenge in the field of breast cancer treatment. However, it is noteworthy that researchers have discovered that the combination therapy of PD-L1 inhibitors with paclitaxel can yield satisfactory outcomes (49). Subsequently, further studies have confirmed that this approach is not restricted to its use with paclitaxel; combinations of doxorubicin or cisplatin with PD-1/PD-L1 inhibitors also represent promising therapeutic strategies for triple-negative breast cancer (50). Similarly, the underlying cause of this phenomenon can also be attributed to alterations in the tumor immune microenvironment (51).

The combination therapy for NSCLC has also become a research focus. A phase III clinical study revealed that, compared to chemotherapy alone, the use of PD-1 inhibitors in conjunction with standard chemotherapy significantly improves the overall survival rate of patients with non-small cell lung cancer (52). The combination immunotherapy involving PD-L1 inhibitors, such as atezolizumab and bevacizumab, has garnered significant attention. When used in conjunction with carboplatin and paclitaxel for the treatment of NSCLC, these therapies have demonstrated notable therapeutic efficacy and are regarded as a first-line treatment option for NSCLC (53–55). The current evidence regarding the enhanced efficacy of combining chemotherapy agents with ICIs in amplifying the antitumor effects of monotherapy remains limited. We propose that this synergy may be attributed to the complementary mechanisms of action between the two treatment modalities. Firstly, chemotherapy agents can promote the release and presentation of tumor antigens, thereby enhancing T cell recognition and attack capabilities against tumors. Concurrently, ICIs serve to restore T cell immune function further. Secondly, chemotherapy can eliminate immunosuppressive cells within the tumor microenvironment, thereby altering it to facilitate a more effective response when ICIs are employed to reinstate immune system functionality.

However, although the combination therapy of chemotherapy agents with PD-1/PD-L1 inhibitors can significantly enhance therapeutic efficacy, issues such as resistance and irAEs persist. Research has indicated that Galectin-3 (Gal-3) plays a role in drug resistance. Numerous patients with lung adenocarcinoma and melanoma who exhibit resistance to PD-L1 inhibitors have been identified, characterized by a high expression of Gal-3 in their tumor cells (56, 57). Gal-3 binding to the glycosylation on PD-L1 can lead to a decreased affinity of atezolizumab for its receptor, resulting in impaired therapeutic efficacy and the development of resistance. However, when the glycosylation is removed from PD-1, the tumor’s sensitivity to the drug is restored (58), and the combined use of GB1211 (a Gal-3 inhibitor) with PD-L1 inhibitors can effectively eliminate Gal-3-induced resistance to pembrolizumab and atezolizumab, thereby enhancing their therapeutic effectiveness (59). The irAEs associated with combination therapy primarily include fever, skin toxicity, thyroid dysfunction, and pneumonia. The incidence of these risks has reached 68.3% (60). Furthermore, some studies have reported that approximately 1.49% of patients experienced fatal outcomes following treatment with platinum-based agents in conjunction with PD-1/PD-L1 inhibitors (61).

It is evident that the combination of PD-1/PD-L1 inhibitors with chemotherapy agents significantly enhances anti-tumor efficacy. However, the cytotoxic effects of chemotherapy drugs still pose a risk of irAEs for patients following treatment. Therefore, further exploration of effective and safe strategies for combined immunotherapy involving PD-1/PD-L1 remains a crucial research direction in cancer therapy.

## Therapeutic strategies involving PD-1/PD-L1 inhibitors in combination with other ICIs

Immune checkpoint markers often play similar roles; therefore, the combination of ICIs is theoretically feasible for enhancing tumor treatment efficacy. CTLA-4 is predominantly expressed on regulatory T cells (Tregs) and activated T cells, where it exerts an inhibitory effect on T cell activation. Currently, it is established that CTLA-4 and PD-1 are co-inhibitory molecules. Evidence suggests that they inhibit T cell activation through distinct mechanisms (62). CTLA-4 binds to CD80 and CD86, thereby utilizing SHP-2 to inhibit downstream signaling induced by TCR and suppressing the PI3K-Akt pathway, which ultimately affects T cell activation (63). Besides, CTLA-4 functions at the early stage of T cell activation, specifically during the “immune initiation phase”, while PD-1/PD-L1 operates during the “effector phase” (64), this indicates that CTLA-4 and PD-1 function at different time points during T cell evolution and may have overlapping effects.

In a clinical trial involving 945 patients with stage III or IV melanoma, the survival rates of patients treated with Ipilimumab (a CTLA-4 inhibitor) and Nivolumab (a PD-1 inhibitor) in combination were investigated and compared to those receiving monotherapy with either drug. The results indicated that the median survival time for the combination therapy group exceeded 60 months, while it was 19.9 months for the Ipilimumab group and 36.9 months for the Nivolumab group. Furthermore, the five-year survival rate in the combination therapy group was also found to be the highest at 52%. However, this study also revealed that the incidence of adverse events was greatest in the combination therapy group (65). Meanwhile, the combination of these two types of ICIs demonstrates a significant advantage over standard targeted therapies or chemotherapy (66, 67). Wei et al. (68) conducted an experiment using the MC38 colon adenocarcinoma mouse model, where they analyzed the T cell phenotypes in tumor samples from mice treated with a combination of CTLA-4 and PD-1 inhibitors through mass cytometry. Their findings concluded that the efficacy of combination therapy is, in most cases, additive to that of monotherapy. Furthermore, they observed an expansion of terminally differentiated effector CD8+ T cells following combination treatment, which also led to an increased frequency of type 1 helper T cells. High-grade neuroendocrine tumors are characterized by more aggressive biological properties and low expression of somatostatin receptors, which often results in a lower response rate to chemotherapy (69). A phase 3 clinical study has demonstrated that 44% of patients with high-grade neuroendocrine tumors exhibit a favorable response to dual blockade therapy targeting CTLA-4 and PD-1. This response rate represents a significant improvement compared to chemotherapy and monotherapy with ICIs. However, the incidence of treatment-related adverse events reached 84.4%, primarily including abnormalities in thyroid and liver function, as well as symptoms such as vomiting and fatigue (70). Although these side effects are considerably milder than the consequences associated with the tumor itself, there remains a pressing need for therapeutic strategies that not only deliver substantial efficacy but also minimize adverse effects in order to enhance patients’ quality of life. However, some clinical trial results have not achieved the expected outcomes. The combination of durvalumab and tremelimumab has shown no significant improvement in treatment efficacy compared to trastuzumab in patients with metastatic urothelial carcinoma and head and neck squamous cell carcinoma (71, 72). In contrast, better therapeutic effects can be observed in patients with non-small cell lung cancer (73), it indicates that the combined immunotherapy strategies involving ICIs still require tailored approaches based on specific tumor subgroups. Furthermore, extensive foundational experiments and clinical research are necessary to develop more specialized treatment guidelines that support these therapeutic regimens.

The efficacy of combining PD-1/PD-L1 inhibitors with other ICIs, such as α-TIM-3, α-LAG-3, α-PVRIG, and α-TIGIT, is currently under investigation. Nivolumab in combination with α-PVRIG or α-TIGIT has demonstrated improved efficacy in patients with PD-L1-positive NSCLC compared to monotherapy combined with placebo groups (74). The efficacy and safety of the combination therapy with α-TIM-3 have been studied across various types of tumors. Research indicates that this combined strategy is well-tolerated by patients, with a slight enhancement in therapeutic effect. However, it is noteworthy that over half of the patients experienced adverse events. Therefore, further research is needed to refine patient selection criteria (75–77). Some scholars have proposed a treatment strategy that combines two types of ICIs with chemotherapy agents. In a mouse model experiment for intrahepatic cholangiocarcinoma, the therapeutic approach involved pre-treatment with a CTLA-4 inhibitor and platinum-based chemotherapy, followed by administration of a PD-1 inhibitor. This combination demonstrated significant survival benefits and lower incidence rates compared to monotherapy with ICIs. Further research revealed that platinum compounds could normalize the vasculature in Intrahepatic Cholangiocarcinoma (ICC), while dual blockade of CTLA-4 and PD-1 resulted in an increased population of CD8+ T cells (78). **Table 2** summarizes the clinical research findings on the combination therapy of PD-1/PD-L1 inhibitors with other ICIs for cancer treatment. The majority of these studies focus on the combination with CTLA-4 inhibitors. This combined therapeutic approach holds promise for application in clinical trials, aiming to address patient resistance to single ICIs.

**Table 2:** The efficacy of PD-1/PD-L1 inhibitors in combination with other types of immune checkpoint inhibitors in cancer treatment.

Combination therapy	Cancer types	Key outcomes						References
		Median Progression-Free Survival (mPFS)	Median Overall Survival (mOS)	Overall Response Rate (ORR)	Disease Control Rate (DCR)	Total TRAEs Rate	≥Grade 3 TRAEs Rate	
PD-1/PD-L1 + CTLA-4 Inhibitors	Colorectal Cancer		6.66 month		49%	94%	44%	(103)
				33%	56%	59%		(104)
	NSCLC			43%			70%	(105)
				30%			29%	(106)
		23.2 month	17.1 month				32.8%	(107)
		2.6 month		33%				(108)
	Hepatocellular Carcinoma	19.53 month		27%		50%	43%	(109)
		2.9 month	9.2 month		47%	41%	19%	(110)
	Melanoma		24.4 month	73%			73%	(111)
		5.0 month	24.7 month			29%	27%	(112)
	Oropharyngeal Cancer			43%			14%	(113)
PD-1/PD-L1 + a-TIM-3 Inhibitors	Microsatellite Instability-High/Mismatch Repair-Deficient Tumors			45.0%	70.0%	65%		(77)
	Solid Tumors			4%	42%			(75)
PD-1/PD-L1 + LAG-3 Monoclonal Antibody	Melanoma	13.3 month				44%	22%	(114)

The blank spaces indicate data that are not mentioned in the references. TRAEs, Treatment-related adverse events.

However, the combinatorial strategies involving ICIs still require extensive research validation. Some studies have reported contrasting conclusions, indicating that the concurrent use of multiple ICIs for cancer treatment may lead to an earlier median onset time of irAEs without a significant enhancement in therapeutic efficacy (79). This suggests that it remains essential to further explore the mechanisms underlying the synergistic effects of ICIs and their roles across different cancer phenotypes.

## TCM in tumor therapy and its comprehensive immunotherapy combined with PD-1/PD-L1 inhibitors

Currently, it is widely recognized that TCM possesses advantages in cancer treatment through multiple pathways and targets. TCM can exert its anti-tumor effects by altering the tumor immune microenvironment and enhancing the immune efficacy of T cells. Additionally, its functions include promoting the proliferation and differentiation of T cells, boosting the activity of natural killer cells, inhibiting immune checkpoints, and modulating immune signaling pathways (80, 81). In TCM, herbal remedies are often utilized as maintenance therapy for patients who have undergone surgery or received radiotherapy and chemotherapy. This approach aims to alleviate adverse reactions following treatment, thereby reducing patient suffering. Additionally, some studies have reported that adjunctive herbal therapy may extend the median survival time of certain patients (82, 83) and decrease the mortality risk associated with cancer (84). Berberine, a compound recognized in TCM, possesses the ability to regulate endocrine metabolism and improve gut microbiota. Additionally, due to its capacity to induce apoptosis in cancer cells, it is frequently employed in the fight against various cancers (85). Research has also indicated that berberine can enhance the sensitivity of tumor cells to CD8+ T cells by reducing the expression levels of PD-L1 on these cells, thereby contributing to immunotherapy efforts (86). As previously mentioned, the homeostasis of gut microbiota plays a crucial role in the body’s ability to eliminate tumor cells. Ginseng Polysaccharides can enhance the sensitivity of tumor patients who are resistant to PD-1 inhibitors by improving the gut microbiota structure in non-small cell lung cancer patients and increasing the microbial metabolite pentanoate (87). The mutations in genes of normal cells disrupt the homeostasis of the extracellular environment, thereby promoting tumor cell proliferation, metastasis, and drug resistance through various signaling pathways. This results in a complex TIME, which is one of the critical factors determining the development and characteristics of tumor cells (88). The complex components of TCM engage multiple signaling pathways, which endows it with unique advantages in cancer treatment. TCM can modulate various cytokines and immune cells within the tumor immune microenvironment TIME, thereby enhancing the body’s immune function (89). Huangqi, a traditional Chinese medicinal herb with a long history, is commonly utilized as one of the herbal components in various bioactive TCM formulas. It has been shown to directly combat tumor cell proliferation and promote apoptosis in tumor cells, thereby reducing tumor volume and inhibiting metastasis. Additionally, Huangqi can enhance immune response by activating macrophages and T cells within the TIME to target and eliminate tumors (90). The Yiyi Fuzi Baijiang Decoction is a TCM formula used for the treatment of gastrointestinal diseases. It has been demonstrated to possess significant efficacy in the management of gastrointestinal tumors. This decoction can alter the gut microbiota structure in ApcMin/+ mice and regulate the Treg/Th17 ratio to control cellular carcinogenesis. Additionally, it inhibits the proliferation and development of intestinal tumor cells in ApcMin/+ mice (91).

TCM emphasizes enhancing the body’s overall immunity to improve its anti-tumor capabilities, which aligns closely with contemporary mainstream concepts of tumor immunotherapy. Additionally, the combined use of TCM and anti-tumor drugs can significantly enhance therapeutic efficacy while reducing side effects, thereby playing a crucial role in improving the survival rates of cancer patients. Numerous studies have reported that TCM can serve as a sensitizer for PD-1/PD-L1 inhibitors by modulating the TIME and improving gut microbiota, thus strengthening anti-tumor immune responses. A meta-analysis conducted an in-depth examination of the effects of TCM combined with PD-1/PD-L1 inhibitors on various tumor subgroups. The findings revealed that, following the combination treatment of TCM and PD-1/PD-L1 inhibitors, there was a significant reduction in both tumor weight and volume compared to treatment with PD-1/PD-L1 inhibitors alone. Additionally, patients experienced a notable extension in survival time, accompanied by a marked increase in the activity of CD4+ T lymphocytes and various growth factors (92). The mice with intestinal tumors treated with a combination of Gegen Qinlian Decoction and PD-1 inhibitors exhibited a significant upregulation of IL-2 levels compared to those treated solely with PD-1 inhibitors. This indicates that the combined therapy is beneficial for restoring T cell function. Furthermore, this study found an increase in the abundance of gut Bacteroides in mice receiving the combined treatment, which can enhance the intestinal immune inflammatory environment and exert immunomodulatory effects by releasing extracellular bacterial DNA (93). The Shenmai injection primarily adjusts the tumor immune microenvironment by inducing the infiltration of NK cell subpopulations. Furthermore, its synergistic effect in combating NSCLC when used in conjunction with PD-1 inhibitors is superior to that of PD-1 inhibitors alone. Ultimately, this combination therapy prolongs the survival time of cancer patients and highlights that NK cell-mediated apoptosis of tumor cells is one of the key synergistic mechanisms underlying this combined treatment approach (94). The combination of demethoxycurcumin and PD-L1 inhibitors can activate the functionality of CD8+ T cells while simultaneously suppressing MDSCs, thereby enhancing the immune defense against tumor cells (95). **Table 3** summarizes several clinical studies on the combination of TCM with PD-1/PD-L1 inhibitors in cancer treatment. However, research in this area remains limited, and most studies have primarily focused on Asian populations. Nevertheless, it is evident from these investigations that the combined use of TCM and PD-1/PD-L1 inhibitors not only demonstrates a favorable anti-tumor effect but also presents a relatively low risk of adverse reactions (even though these data are derived from small sample sizes). This underscores the need for further in-depth exploration into the integration of traditional and modern medical approaches for cancer treatment. **Table 4** provides a brief introduction to the traditional Chinese medicine mentioned above.

**Table 3:** The efficacy of traditional Chinese medicine combined with PD-1/PD-L1 inhibitors in the treatment of cancer.

Cancer types	TCM	Key outcomes			TRAEs rates			Total TRAEs rates	References
		Overall Response Rate (ORR)	Disease Control Rate (DCR)	median Progression-Free Survival (mPFS)	Digestive system	Circulatory system	Nervous system		
NSCLC	Yifei Fuzheng Anti -Tumor Decoction	67.86%	96.43%		10.72%	14.29%			(115)
	Hengyuan Capsule	55%	85%		20%	25%			(116)
	Sha Shen Mai Dong Soup	52.1%	85.4%		14.6%	27.2%			(117)
	Compound Kushen Injection	53.33%	93.33%			70%	76.67%		(118)
	Compound Kushen Injection	64%	76%		10%	10%			(119)
Esophageal Cancer	Bazhen Huaji Decoction	57.69%	90.38%		9.62%	1.92%	1.92%	13.46%	(120)
Epithelial Ovarian Cancer	Huangqi Zhishi Decoction	91.1%			6.7%	4.44%	2.22%	17.78%	(121)
Metastatic Colorectal Cancer	Huangci Granule			9.59 month	45%	25%	13%		(122)

The blank spaces indicate data that are not mentioned in the researches. TRAEs: Treatment-related adverse events. The TRAEs of the digestive system mainly include nausea, vomiting, loss of appetite, constipation, etc.; the TRAEs of the blood system mainly include reduction of red blood cells, white blood cells, and platelets, etc.; the TRAEs of the nervous system mainly include dizziness, neurotoxic reactions, etc.

**Table 4:** A brief description of the TCM mentioned in this review.

Traditional Chinese Medicine	Primary Chemical Monomer Components	Primary efficacy	Notes
Berberine	Quaternary Ammonium Alkaloids	Antibacterial, blood glucose-lowering, lipid-lowering, anti-inflammatory, and antitumor properties	Originating from botanical sources, including Coptis chinensis and Phellodendron amurense
Astragalus	Astragaloside (saponin), calycosin (flavonoid), and astragalus polysaccharides	Immunomodulation, antioxidant activity, cardiovascular protection, blood glucose/lipid reduction, renal protection	
Ginseng Polysaccharides	Arabinan	Immunomodulation, fatigue resistance, enhancement of intestinal immunity, and adjuvant therapy for tumor suppression	The content of ginseng root accounts for 10–20% of its dry weight
Yiyi Fuzi Baijiang Decoction	Oleanolic Acid, Ursolic Acid, Caffeic Acid, Luteolin	Antitumor, antioxidant, anti-inflammatory, and protection of red blood cells against oxidative damage	The formulation includes three medicinal ingredients: Coix seed, Aconite, and Patrinia herb
Gegen Qinlian Decoction	Puerarin, Baicalin, Berberine, Glycyrrhizic acid	Fever reduction, diarrhea relief, anti-inflammatory action, and intestinal mucosal repair	
Shengmai Injection	Ginsenosides, Ophiopogon saponins	Anti-shock, improvement of heart failure, protection against ischemia-reperfusion injury, and immune modulation	The formulation contains red ginseng and Ophiopogon japonicus. Intravenous administration requires dilution

The combination of TCM with PD-1/PD-L1 inhibitors in comprehensive immunotherapy offers new perspectives and strategies for cancer treatment. Existing research indicates that TCM, through its multi-component and multi-target mechanisms, can modulate the tumor microenvironment, enhance immune cell functionality, and exert a synergistic anti-tumor effect when used alongside PD-1/PD-L1 inhibitors. However, several issues remain to be addressed urgently, such as the identification of specific active components in TCM, a deeper elucidation of their mechanisms of action, and the establishment of standardized protocols for combination therapy. Nevertheless, with the ongoing advancement in integrative studies between Western and TCM, this comprehensive immunotherapy holds promise for providing new therapeutic hope to more cancer patients and advancing the field of tumor immunotherapy further.

## Perspective and conclusion

PD-1/PD-L1 inhibitors represent a revolutionary class of drugs in tumor immunotherapy, demonstrating significant efficacy across various malignancies. However, monotherapy still faces challenges such as low response rates, resistance, and irAEs, the existing combination strategies continue to face a dual challenge of efficacy and toxicity: the combination of these inhibitors with chemotherapy agents or multiple ICIs has been shown to enhance therapeutic efficacy to some extent and has become the preferred treatment option for certain tumors; nevertheless, issues related to resistance and irAEs remain unresolved and may potentially increase toxic side effects. In contrast to chemotherapy agents, TCM is characterized by its multi-component, multi-targeted approach that promotes holistic regulation while causing minimal damage to normal cells. TCM can exert unique advantages in anti-tumor activity and immune modulation. Recent studies have increasingly indicated that certain herbal medicines can synergistically enhance the anti-tumor effects of PD-1/PD-L1 inhibitors through mechanisms such as modulating the tumor microenvironment, enhancing immune cell function, and inhibiting PD-1/PD-L1 expression.

This study not only summarizes the use of PD-1/PD-L1 inhibitors in combination with other Western medical approaches for cancer treatment but also conducts an in-depth review of relevant research on their concurrent application with TCM. The aim is to explore the feasibility of combined anti-tumor therapies from a TCM perspective. Traditional Chinese medicine can reshape the immune microenvironment through multi-target mechanisms, reduce irAEs, and optimize the anti-tumor effects of ICIs. Numerous herbal formulas and active components, such as astragalus polysaccharides and cordycepin, have been shown to modulate the tumor immune microenvironment through multi-target actions: promoting dendritic cell maturation, enhancing T/NK cell functions, inhibiting regulatory T cells/myeloid-derived suppressor cells activity, downregulating immunosuppressive factors (such as TGF-β and IL-10), and even directly or indirectly regulating PD-1/PD-L1 expression. These mechanisms hold promise for reversing immunosuppressive states and enhancing the efficacy of PD-1/PD-L1 inhibitors (96–99). Compared to chemotherapy or dual immunotherapy, traditional Chinese medicine demonstrates unique and significant advantages in terms of “toxicity reduction.” Clinical practices and some studies have indicated that traditional Chinese medicine can alleviate toxic reactions such as rashes, diarrhea, pneumonia, and hepatitis. Additionally, it has the potential to improve overall patient symptoms (such as fatigue, loss of appetite, and sleep disturbances), thereby enhancing quality of life and treatment tolerance (100–102).

However, it is essential to recognize that there are significant deficiencies in the current research on the combination of traditional Chinese medicine with PD-1/PD-L1 inhibitors for cancer treatment. The majority of existing evidence stems from small-sample retrospective analyses, case reports, or basic research, lacking rigorously designed and large-sample prospective randomized controlled trials (RCTs) that can provide high-level evidence in evidence-based medicine. Looking ahead, it is crucial to elucidate the underlying mechanisms of action when combining PD-1/PD-L1 inhibitors with TCM. Identifying and optimizing the best combinations of these therapies will be essential. Conducting high-quality clinical research will provide a comprehensive assessment of the safety and efficacy of combination regimens while further exploring the potential for integrating TCM with various ICIs in cancer treatment. This endeavor holds promise as an important area for leveraging TCM in anti-tumor strategies.
